# Development and analytical validation of a multiplex diagnostic qPCR-array as a potential application in predicting the response to neoadjuvant chemotherapy in muscle invasive bladder cancer

**DOI:** 10.1016/j.tranon.2025.102528

**Published:** 2025-09-09

**Authors:** Toru Sakatani, Sunao Tanaka, Kaoru Murakami, Francisco Aguilar, Owen T.M. Chan, Catherine Bresee, Daniel J. Luthringer, Charles J. Rosser, Wayne Hogrefe, Hideki Furuya

**Affiliations:** a Samuel Oschin Comprehensive Cancer Institute, Cedars-Sinai Medical Center, Los Angeles, CA, USA; b Department of Urology, Kyoto University, Kyoto, Japan; c Clinical and Translational Research Program University of Hawaii Cancer Center, Honolulu, HI, USA; d Biostatistics Shared Resources, Cedars-Sinai Medical Center, Los Angeles, CA, USA; e Department of Pathology, Cedars-Sinai Medical Center, Los Angeles, CA, USA; f Department of Urology, Cedars-Sinai Medical Center, Los Angeles, CA, USA; g Nonagen Bioscience Corp., Los Angeles, CA, USA; h Department of Biomedical Sciences Cedars-Sinai Medical Center, Los Angeles, CA, USA

**Keywords:** Molecular diagnostic, Muscle invasive bladder cancer, Neoadjuvant chemotherapy, Multiplex

## Abstract

Bladder cancer (BC) remains a common malignancy, with muscle-invasive bladder cancer (MIBC) comprising 20 % of cases and a poor 5-year survival rate of ∼50 %. While neoadjuvant chemotherapy (NAC) followed by radical cystectomy is the standard treatment for locally advanced disease, NAC is limited by toxicity and non-response in many patients. Predictive biomarkers are urgently needed to guide treatment decisions. We developed and analytically validated a multiplex qPCR array to measure mRNA expression of 10 NAC response-associated biomarkers in MIBC. RNA from 8 formalin-fixed paraffin-embedded (FFPE) and 4 fresh-frozen (FF) MIBC specimens was analyzed to assess the effects of tissue type, necrosis, and pre-analytical variables on assay performance. The assay showed high concordance between FF and FFPE samples, with stable performance using FFPE curls stored at ≤4 °C for up to two weeks. It remained robust across RNA input levels (5–100 ng) and required a minimum quality threshold (DV200 >15 %). Necrosis had minimal impact on gene expression, and reproducibility was confirmed across technicians and time points. In conclusion, this qPCR array-based assay offers a reliable platform for evaluating key biomarkers in FFPE tissues and holds promise as a potential clinical tool to predict NAC response in MIBC patients.

## Highlights

• A multiplex qPCR assay was developed for 10 NAC-response genes in bladder cancer.
• The assay works reliably on both FFPE and fresh-frozen tissue specimens.
• Robust performance was observed across RNA quality, quantity, and storage conditions.
• Gene expression was stable even in necrotic samples and across different technicians.
• The assay supports precision medicine by predicting NAC response in MIBC patients.

## Introduction

Bladder cancer (BC) is the second most common genitourinary malignancy in the world, the sixth most common cancer in men and the 17th most common cancer in women [1]. Muscle-invasive bladder cancer (MIBC) represents approximately 20 % of newly diagnosed cases of BC [2], and is characterized by an overall poor prognosis with a 5-year overall survival (OS) of ∼50 % [3]. Although radical cystectomy (RC) remains the most effective treatment for MIBC, a half of MIBC treated with RC eventually recur with distant metastases and fatal outcome [2].

Previous studies have demonstrated that neoadjuvant chemotherapy (NAC) followed by RC increases the probability of downstaging BC prior to cystectomy and is associated with improved survival [3,4]. In addition, a recent meta-analysis of 15 randomized NAC clinical trials noted an improved cancer-specific survival by 5–10 % compared to no NAC [5]. With the accumulating evidence, currently AUA/ASCO/SUO Guideline for non-metastatic MIBC strongly recommends offering cisplatin-based NAC to eligible RC patients [6]. However, NAC has the potential risk that a segment of patients do not respond, suffer treatment toxicity, and experience critical surgical delays [7]. AUA/ASCO/SUO Guideline noted that there are no validated predictive factors or clinical characteristics (including age) associated with an increased or decreased probability of response and benefit using cisplatin-based NAC. Therefore, more precise stratification of patients with indications for NAC is urgently needed to identify patients more likely to respond, and transition those who are likely to fail to other strategies.

Our prior studies have identified a panel of 10 urine-based protein biomarkers including Alpha-1 Antitrypsin (A1AT), angiogenin (ANG), Apolipoprotein E (APOE), Carbonic Anhydrase 9 (CA9), Interleukin-8 (IL8), matrix metalloproteinases 9 (MMP9), MMP10, plasminogen activator inhibitor-1 (PAI1), syndecan-1 (SDC1), and vascular endothelial growth factor A (VEGFA), which are significantly associated with BC [8]. Previous studies using immunostaining have shown that these 10 protein biomarkers are highly expressed in neoplastic urothelium compared to benign urothelium, and higher levels of these biomarkers are associated with more aggressive BC [9]. Additionally, we noted that mRNA expression of these 10 biomarkers to have prognostic power with a large gene expression cohort from The Cancer Genome Atlas (TCGA) [10]. In a separate analysis of the GSE87304 cohort (Gene Expression Omnibus; GEO), in which all patients received neoadjuvant chemotherapy (NAC), we further demonstrated that combined expression of these biomarkers was significantly associated with recurrence-free survival [10]. Taken together, these findings suggest that the 10-gene panel may be biologically relevant to NAC response, and thus the gene expression profile of the 10-gene panel may also have predictive value for determining response to NAC. This concept is further supported by emerging studies demonstrating that molecular biomarkers and gene expression signatures can inform NAC sensitivity and guide personalized treatment strategies in muscle-invasive bladder cancer [[11], [12], [13], [14]]. However, although several predictive gene expression signatures have been proposed, none have yet been widely adopted into clinical practice, largely due to issues with reproducibility, lack of analytical validation, and limited feasibility for routine clinical use. Furthermore, most existing signatures rely on tumor tissue obtained from cystectomy specimens, rather than pre-treatment biopsies or non-invasive approaches. Our multiplex qPCR-array platform, designed to assess a focused panel of analytically validated biomarkers, offers the potential for a clinically practical, minimally invasive, and reproducible tool to predict NAC response prior to definitive surgery. Nevertheless, a rigorous and validated assessment of gene expression levels within this biomarker panel must first be established.

In this study, we developed a custom multiplex qPCR array-based assay to evaluate the mRNA expression of our bladder cancer associated diagnostic signature and analytically validated its performance using FFPE of surgical specimens obtained from MIBC based on the Clinical and Laboratory Standards Institute (CLSI) guidelines [15]. The main objective of this study was to perform analytical validation of the assay—specifically, to optimize its components into an integrated workflow and establish a standard operating protocol for sample collection, processing, quality control, and data generation. While the number of specimens analyzed was modest (8 FFPE and 4 fresh-frozen samples), this sample size is appropriate for analytical validation studies, which are designed to assess technical robustness and reproducibility before advancing to larger-scale clinical validation. Here, we provide an overview of the analytical performance of the multiplex qPCR array-based assay (Nexus-Dx™, Nonagen Bioscience, Los Angeles, CA).

## Materials and methods

### Study design

**Fig. 1:**
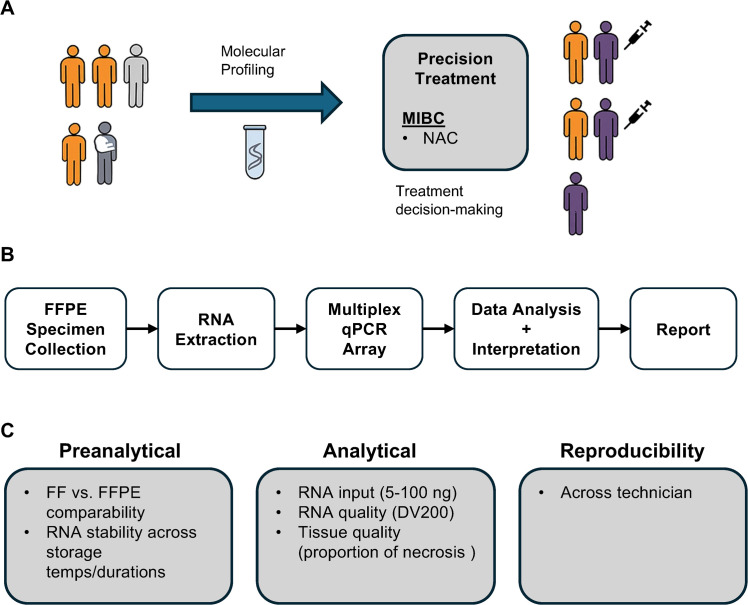
Overview of the assay. **A)** The assay relies on a multiplex qPCR array platform to simultaneously measure gene expression of 10 transcripts associated with BC. **B)** The workflow consists of a first evaluation of slides by a pathologist, RNA extraction, followed by target pre-amplification and a multiplex qPCR array. qPCR data are processed through the algorithm with reporting. **C)** Performance is evaluated for preanalytical and analytical parameters followed by a reproducibility study.

Assay performance was assessed by studying various pre-analytical and analytical factors, focusing on reproducibility, sensitivity, accuracy, and specificity using MIBC surgical specimens from patients before NAC (Fig. 1).

### Specimens

To evaluate the analytical performance of the assay, we employed 8 Formalin-Fixed Paraffin-Embedded (FFPE) specimens and 4 fresh-frozen (FF) tissues from advanced BC patients stored in the Department of Pathology at Cedars-Sinai Medical Center. This study was approved by Cedars-Sinai IRB (IRB# STUDY00000451) according to institutional policy for human subject research. Specimens included the surgical specimen of transurethral resection of bladder tumor (TURBT) and the RC performed from 2016 to 2020.

### RNA extraction

Four FFPE curls of 10 μm thickness were cut per FFPE block and total RNA was extracted using AllPrep DNA/RNA FFPE Kit (Cat# 80,234, Qiagen, Hilden, Germany) according to the manufacturer’s instructions after the deparaffinization steps using xylene. RNA was eluted in 30 μL of water and their yield and quality were evaluated by BioAnalyzer (RNA Pico or Nano, depending on RNA yield) and expressed as DV200, which evaluates the percentage of fragments of >200 nucleotides.

### cDNA synthesis

cDNA synthesis was conducted using 5 μL of RNA solution and Prelude One-Step PreAmp master mix (Cat# 638,554, Takara Bio Inc., Japan), with 5 μL of the primer pool for 13 genes including 10 biomarkers genes (ANG, APOE, A1AT, CA9, IL8, MMP9, MMP10, PAI1, SDC1, and VEGFA) and the 3 reference genes (TBP, ATP5E and CLTC) for normalization of Quantitative PCR (qPCR) data as well as RT control primer (BIO-RAD, Cat #10,025,695). The preamplification assay was performed at cycling conditions of 42 °C 10 min, 95 °C 2 min, 14 cycles of 95 °C 10 s and 60 °C 4 min, and 4 °C hold.

### Primer and PCR array design

Primers for 10 biomarkers genes and 3 reference genes were designed using Primer-BLAST tools according to the desired gene target. The sequences for the target genes were obtained from GenBank within NCBI. Exon/intron boundary selection was not specifically performed; however, RNA samples were DNase-treated prior to reverse transcription to minimize genomic DNA contamination according to instructions from Qiagen AllPrep DNA/RNA FFPE Kit. Some candidate primers obtained then underwent further selection such as selection in forward and reverse primer temperatures <5 °C, % GC values at 40–60 % and no self-3-complementarity values. To ensures reliable performance across samples, we confirmed that all primers showed amplification efficiencies between 90 % and 110 %. The specificity of this primer was tested using Standard Nucleotide BLAST. The custom PCR array was designed on Bio-Rad PrimePCR Tools and the array in a 384-well plate was produced by Bio-Rad. To ensure technical performance and data quality, the array included three built-in assay controls: PrimePCR DNA Contamination Control Assay (to monitor gDNA contamination), PrimePCR Positive Control Assay (to assess qPCR efficiency), and PrimePCR Reverse Transcription Control Assay (to verify RT reaction success).

### Real-time quantitative PCR array

The RT-qPCR analyses were performed in the Applied Biosystems QuantStudio 6 Flex Real-Time PCR system (Thermo Fisher Scientific, Waltham, MA, USA) using PowerUP SYBR Green master mix for qPCR (Thermo Fisher Scientific). One reaction (10 µl total per well) contained: 5 µl of PCR master mix, 1 µl of cDNA template and each primer dried in well. The PCR program was set as follows: 95 °C for 10 s (denaturation), followed by 40 cycles of denaturation at 95 °C for 5 s, annealing and extension at 60 °C for 30 s, followed by melting curve read from 60 °C to 95 °C with increment 0.2 °C every 10 s.

### Histologic evaluation of H&E

Two board-certified anatomic pathologists (O.T.M.C. & D.J.L.) reviewed H&E slides from each FFPE block to evaluate histology and document the approximate percentage of necrotic cells of total tumor area.

### Statistical analysis

We calculated RNA expression level based on 2^-ΔCt as described previously which were log-transformed prior to analysis[16]. Separate mixed regression models were used to test for differences across each of the independent processing factors on the dependent factor of overall RNA expression levels. Each processing factor was modeled independently as the fixed effect, with random effects of each gene clustered by subject (e.g. a 2-level hierarchical design), along with an unstructured covariance matrix. Residuals were inspected to confirm appropriateness of modeling. Differences were considered significant where *p* < 0.05. Data analysis performed using SAS v9.4 software.

## Results

### Preanalytical validation

#### RNA stability

For RNA analysis, FF tissues are considered as the “gold standard” in basic science research. In the real world, FFPE samples are widely used for biological analysis because of their more pristine histology which can lead to more accurate diagnoses. However, RNA derived from FFPE tends to be degraded, chemically modified, and cross-linked due to fixation and archiving methods, affecting the performance of the molecular analysis such as RNA sequence or qPCR [17]. We assessed the effect of FFPE processing compared to FF on the detection of the bladder cancer associated diagnostic signature using qPCR array assay.

**Fig. 2:**
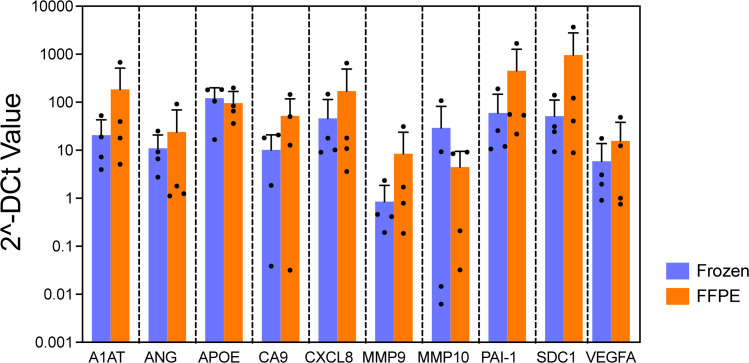
RNA stability test: Comparison of bladder cancer associated diagnostic signature expression pattern between Frozen tissue and paired FFPE. Bar graph shows gene expression level, indicating the expression of each gene derived frozen tissue on left and FFPE on right in same color bar per gene. No difference was observed in gene expression patterns between frozen tissue and paired FFPE (*p* = 0.13, mixed model regression). Error bars represent SD.

First, 4 FF tissues were bisected. One piece was stored at −80 °C and the other was processed to generate FFPE blocks according to College of American Pathologists Guidelines [18]. For the FFPE blocks, the bisected FF tissues were fixed in 10 % neutral buffered formalin for 24 h and immersing them in 70 % ethanol. Then, they were processed in a Leica ASP300 tissue processor (Leica Microsystems, Buffalo Grove, IL) within 7 days. Next, we compared qPCR data using RNA isolated from FFPE blocks with matched FF tissues as controls. No statistically significant difference was found between the FFPE and the FF tissues on gene expression pattern (Fig. 2; *p* = 0.13). The finding demonstrated that qPCR profiling on archival FFPE blocks can generate the reliable data for assessing RNA expression of the bladder cancer associated diagnostic signature.

#### Specimen format

FFPE specimens are often received as unstained slides in an actual clinical situation. Transport and storage environments can adversely affect samples leading to degradation of RNA, which could alter experimental results. To address this issue, we compared the expression pattern of the BC associated diagnostic signature among the FFPE curl stored at various temperatures and durations as follows. Two FFPE blocks (High quality FFPE containing >80 % viable cells and low quality FFPE containing <25 % viable cells) were cut as 4 curls of 10 μm per condition and stored at room temperature (RT), 4 °C, −20 °C, and −80 °C. RNA was extracted on 1, 7, and 14 days of storage, cDNA was immediately synthesized. Finally, PCR array assay was performed, and the bladder cancer associated diagnostic expression profiles were compared.

**Fig. 3:**
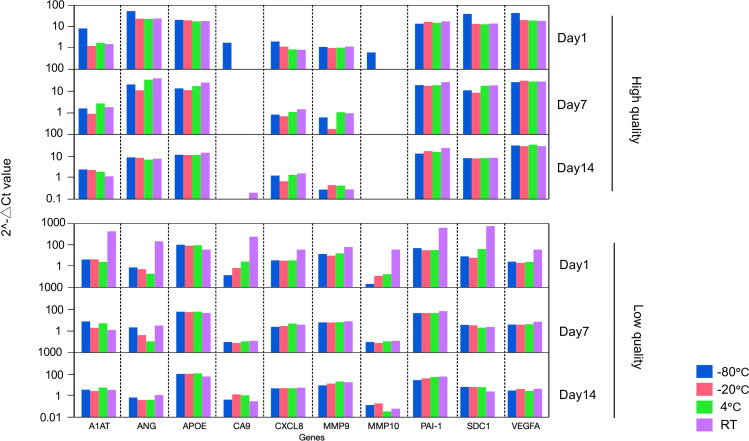
Specimen format test: Comparison of bladder cancer associated diagnostic signature gene expression pattern at various days and storage temperature. Day indicates the date when RNA was extracted from FFPE curl storage at RT, 4 °C, −20 °C, and −80 °C. One high quality and one low quality FFPE were used.

This study revealed that, for all conditions, a similar gene expression pattern was demonstrated across any storage temperature and periods, except for FFPE blocks processed at day 1 and stored at RT (Fig. 3, Table 1). The result supports that the quality of the RNA in the assay can be ensured if the FFPE sample is stored at or below 4 °C and performed within 2 weeks of its sectioning after the from blocks.

**Table 1:** Summary of samples status for specimen format test.

Day of RNA extraction	Storage temperature ( °C)	High % viable cells		Low % viable cells	
		RNA concentration (ng/μl)	DV200( %)	RNA concentration (ng/ μl)	DV200( %)
1	−80	134	10	81	27
	−20	185	42	43	23
	4	235	42	57	12
	RT	219	45	22	10
7	−80	303	38	71	19
	−20	208	40	74	23
	4	270.4	35	85	30
	RT	274	43	72	30
14	−80	268	40	58	24
	−20	238	40	68	24
	4	256	41	48	25
	RT	215	41	89	33

### Analytical validation

#### RNA input

In clinical practice, it is expected that slight discrepancies in the amount of RNA recovered will occur due to variations in tissue size, fixation process, FFPE storage methods, and age of FFPE blocks. To evaluate the minimum amount of RNA input to accurately profile the bladder cancer associated diagnostic signature gene expression, we used 2 FFPEs with high (>80 %) and 2 FFPEs with low (<25 %) % viable cells to evaluate the consistency of the gene expression among various input amounts.

**Fig. 4:**
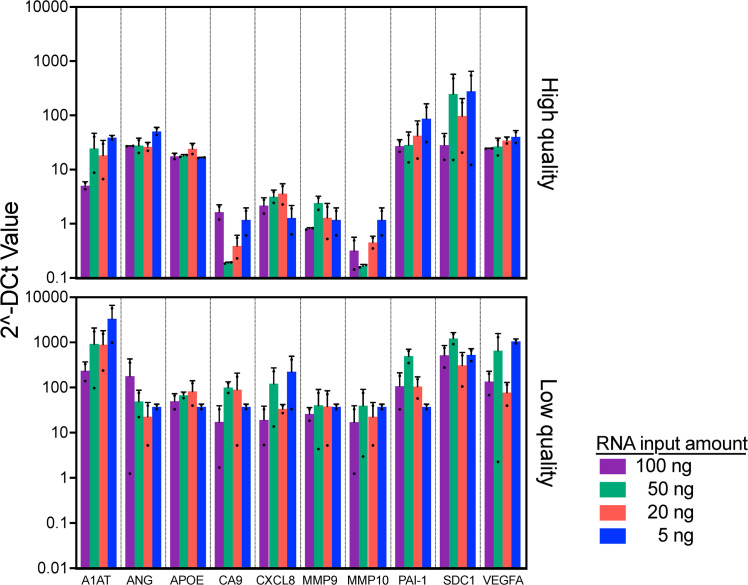
RNA input test: Comparison of bladder cancer associated diagnostic signature gene expression among different RNA input amount. Two samples on the top have highest quality FFPE and the other 2 samples on the bottom have lowest quality FFPE among current cohort were used. Significant difference in overall expression levels between high- and low-quality *p* < 0.01, mixed model regression. Error bars represent SD.

First, we obtained high quality (DV200 of 29 and 33 %) and quantity (concentrations of 173 and 297 ng/μl) of RNA from high quality FFPE blocks, while RNA quality and quantity from low quality FFPE blocks are low as DV200 of 15 and 25 %, and concentrations of 23 and 47 ng/μl, respectively. With the RNA samples, we evaluated the gene expression pattern. The similarity of gene expression pattern was observed among high quality FFPE blocks across all input amount of RNA (5–100 ng, *p* = 0.27). On the other hand, we noted a discrepancy in the expression patten from the low quality FFPE blocks (Fig. 4, *p* = 0.025). Specifically, the gene expression from the low quality FFPE blocks demonstrated higher ΔCt values. Because we added the same amount of RNA, the viable RNA amount ( %DV200 x RNA amount) should not be too different. Thus, we consider that the difference in ΔCt values between high and low quality FFPE blocks may be due to tumor status, such as grade, stage, and/or aggressiveness. Taken together, the results suggest that the assay was robust enough when RNA quality/quantity are above DV200 of 15 % and 5 ng.

#### Tissue quality

**Fig. 5:**
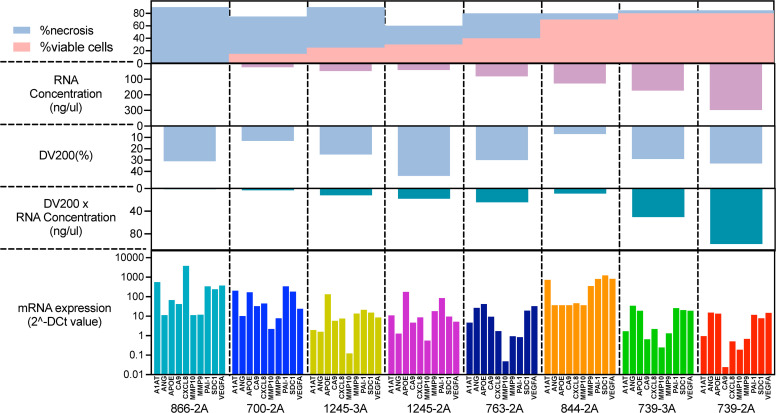
Tissue quality test: Association of proportion of necrosis in specimen and bladder cancer associated diagnostic signature gene expression. Top of the graph shows the proportion of necrosis as blue area and viable cells as pink area. The green graph bars on the middle shows RNA quality as DV200 ( %) multiplied with RNA concentration suggesting the amount of RNA which is available for analyzation. There is an association of the proportion of necrosis and RNA quality. The bottom of the graph indicating that gene expression is still detectable even in the sample with highest proportion of necrosis.

The proportion of necrosis can vary widely not only between different cases but also within the same case, which can affect the reliability of gene expression results. First, we observed a tendency for the proportion of necrosis to be inversely correlated with the yield of extracted RNA (Fig. 5). On the other hand, the RNA quality (DV200) evaluated by Bioanalyzer was not correlated with the proportion of necrosis, suggesting that the RNA quality may be due to other factors, such as tissue size, fixation process, FFPE storage methods, and age of FFPE blocks. We also observed consistency in gene expression in the pair of FFPE blocks within the same case with different necrosis rates, which are the pairs of 739–3A/739–2A and 1245–3A/1245–2A. Collectively, these results suggested that our assay could tolerate the presence of tissue necrosis. Though, the sample with 90 % of necrosis showed the gene expression of the bladder cancer associated diagnostic signature as well as other samples with low proportion of necrosis, the best performance is seen when there is <50 % necrosis present.

### Reproducibility

**Fig. 6:**
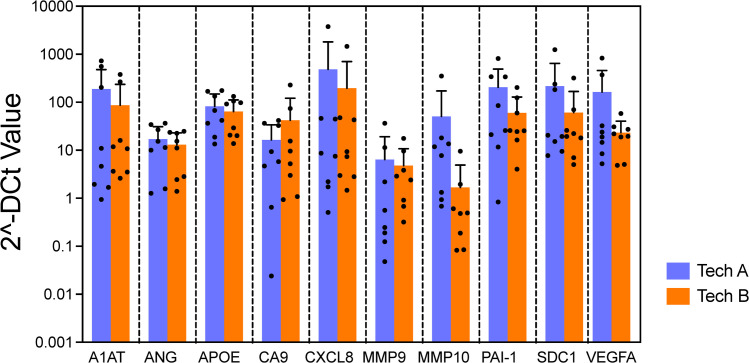
Reproducibility test: Verification of the Reproducibility of Assay Results by Two Laboratory Technicians. No difference was observed in gene expression levels obtained from the qPCR array for all genes across two different technicians (*p* = 0.051, mixed model regression). Error bars represent SD.

To evaluate the reproducibility and robustness of this assay, the bladder cancer associated diagnostic signature gene expression pattern from 2 independent technicians using the same 8 FFPE samples were compared. Multiplex qPCR assays were performed on all samples using a single plate on different dates by each technician. The results from two technicians demonstrated strong correlation (R^2^=0.82), suggesting high reproducibility of the gene expression pattern (Fig. 6). This assists in ensuring high accuracy of the assay despite variations in experimental conditions and days within an actual clinical setting.

## Discussion

Currently, NAC followed by RC is the gold standard treatment for the patients with locally advanced BC. Patients with NAC failure generally have a poorer prognosis when compared to those who achieve significant tumor downstaging [19]. Because there are no validated predictive factors or clinical characteristics associated with an increased or decreased probability of NAC response, there is an urgent unmet need for effective and accurate test that predicts response to NAC. In this study, we outlined the development and analytical validation of a qPCR array-based assay that may predict clinical response to cisplatin-based NAC. The entire process including specimen collection, processing, qPCR, and the data analysis was evaluated to determine robustness across numerous sample features, including histologic status. All of these parameters passed our internal quality assurance plan. We recognize that the cohort size is limited. However, this study was designed specifically for analytical validation to assess assay reproducibility and performance under variable conditions. Similar studies in the field have relied on comparable sample sizes for this stage of development [[20], [21], [22], [23], [24]]. A prospective clinical validation cohort is being pursued to evaluate predictive accuracy in patients undergoing NAC. Of note, the intent of the qPCR array-based diagnostic assay is to measure the relative abundance of mRNA associated with a bladder cancer associated diagnostic signature that we have reported [10].

The overall validation workflow for the assay utilized a series of samples prepared in different conditions to determine robustness, precision, accuracy, and potential confounders. From this evaluation, multiple quality controls (QCs) obtained from the manufacturer, such as gDNA, PCR, and RT, were included in the PCR array plate and they will serve as daily QC parameters. Specifically, the QC measures for gDNA, PCR, and RT were used to monitor genomic DNA contamination, PCR quality, and reverse transcription quality, respectively. The bioinformatics pipeline ensures quick data analysis, delivering results in less than five days. The analytical validation studies reported here demonstrated that ideal specimen characteristics minimally affect the qPCR array-based diagnostic test. First, we confirm that the results from FFPE RNA showed equivalent to RNA isolated from FF tissues. Then we evaluated the effect of storage condition after sectioning FFPE blocks. The results show that assay accuracy is maintained when samples are stored at or below 4 °C and tested within 2 weeks after sectioning curls from FFPE blocks. Next, we evaluated the effects of quality and amount of RNA samples on the test results. To evaluate the minimum amount of RNA input, we employed RNA samples with high and low quality. The results suggest the assay can handle as low as DV200 of 15 % and 5 ng, while the ideal DV200 is >30 %. Interestingly, the amount of RNA was associated with % of viable tumor, while the quality of RNA ( % DV200) was not. Therefore, the specimens should not have >80 % necrosis present to obtain sufficient amount of RNA for qPCR array test. Finally, we demonstrated consistent gene expression patterns by different technicians on different days, thus attesting to the assay’s reproducibility.

It is also important to consider the role of prognostic factors across bladder cancer subtypes. For NMIBC, prognostic assessment is typically based on tumor grade, stage, and recurrence/progression risk stratification models, whereas in MIBC, prognosis is more strongly linked to molecular subtypes and systemic treatment responsiveness. Recent reviews emphasize both shared and distinct prognostic features between NMIBC and MIBC [25,26]. Future clinical validation studies of our assay will benefit from incorporating such comparative perspectives to refine its predictive and translational value across the disease spectrum.

## Conclusions

In conclusion, this analytical validation study identified optimal sample preparation and storage conditions for the multiplex qPCR array-based assay, ensuring the integrity and reliability of results across varying conditions. Additionally, we determined the minimum quality and quantity of samples necessary for accurate and reproducible testing. These findings are critical for standardizing the use of the multiplex qPCR array-based assay in clinical settings and maximizing its diagnostic performance. With these optimized parameters in place, the multiplex qPCR array-based assay is well-positioned for further clinical validation and potential widespread use in BC. Future studies will be needed to confirm these findings in diverse patient populations and real-world conditions.

## List of abbreviations

Bladder cancer (BC) muscle-invasive bladder cancer (MIBC) neoadjuvant chemotherapy (NAC) formalin-fixed paraffin-embedded (FFPE) fresh-frozen (FF) overall survival (OS) radical cystectomy (RC)

The Cancer Genome Atlas (TCGA)

Gene Expression Omnibus (GEO)

Transurethral resection of bladder tumor (TURBT)

Quantitative PCR (qPCR) room temperature (RT)

## Ethics approval and consent to participate

This study received approval and a waiver of consent to use previously banked de-identified urine samples from the Cedars-Sinai Medical Center Institutional Review Board, Los Angeles, CA (IRB # STUDY00000451). Study performance complied with the tenets of the Declaration of Helsinki.

## Consent for publication

Not applicable.

## Availability of data and materials

The primer sequence, anonymized datasets used and/or analyzed during the current study are available from the corresponding author upon reasonable request.

## Funding

This work was supported by research grants UH3 CA271377 (CJR), R01 CA277810 (HF/CJR), U54 CA274375 (HF/CJR) and R01 CA198887 (CJR).

## CRediT authorship contribution statement

**Toru Sakatani:** Writing – original draft, Visualization, Methodology, Formal analysis, Data curation. **Sunao Tanaka:** Data curation. **Kaoru Murakami:** Data curation. **Francisco Aguilar:** Data curation. **Owen T.M. Chan:** Data curation. **Catherine Bresee:** Visualization, Formal analysis, Data curation. **Daniel J. Luthringer:** Resources, Data curation. **Charles J. Rosser:** Writing – review & editing, Supervision, Resources, Investigation, Funding acquisition, Conceptualization. **Wayne Hogrefe:** Writing – review & editing, Investigation. **Hideki Furuya:** Writing – review & editing, Writing – original draft, Supervision, Project administration, Methodology, Investigation, Funding acquisition, Formal analysis, Data curation, Conceptualization.

## Declaration of competing interest

The authors declare the following financial interests/personal relationships which may be considered as potential competing interests:

Charles J. Rosser and Wayne Hogrefe are officers at Nonagen Bioscience. The other authors report no potential conflicts of interest, financial or otherwise.
